# The Regulation of Plant Vegetative Phase Transition and Rejuvenation: miRNAs, a Key Regulator

**DOI:** 10.3390/epigenomes5040024

**Published:** 2021-10-18

**Authors:** Tajbir Raihan, Robert L. Geneve, Sharyn E. Perry, Carlos M. Rodriguez Lopez

**Affiliations:** 1 Environmental Epigenetics and Genetics Group, Department of Horticulture, University of Kentucky, Lexington, KY 40546, USA; tajbi@uky.edu; 2 Department of Horticulture, University of Kentucky, Lexington, KY 40546, USA; rgeneve@uky.edu; 3 Department of Plant and Soil Sciences, University of Kentucky, Lexington, KY 40546, USA; sperr2@uky.edu

**Keywords:** rejuvenation, phase change, epigenetics, vegetative

## Abstract

In contrast to animals, adult organs in plants are not formed during embryogenesis but generated from meristematic cells as plants advance through development. Plant development involves a succession of different phenotypic stages and the transition between these stages is termed phase transition. Phase transitions need to be tightly regulated and coordinated to ensure they occur under optimal seasonal, environmental conditions. Polycarpic perennials transition through vegetative stages and the mature, reproductive stage many times during their lifecycles and, in both perennial and annual species, environmental factors and culturing methods can reverse the otherwise unidirectional vector of plant development. Epigenetic factors regulating gene expression in response to internal cues and external (environmental) stimuli influencing the plant’s phenotype and development have been shown to control phase transitions. How developmental and environmental cues interact to epigenetically alter gene expression and influence these transitions is not well understood, and understanding this interaction is important considering the current climate change scenarios, since epigenetic maladaptation could have catastrophic consequences for perennial plants in natural and agricultural ecosystems. Here, we review studies focusing on the epigenetic regulators of the vegetative phase change and highlight how these mechanisms might act in exogenously induced plant rejuvenation and regrowth following stress.

## 1. Introduction

Plant development is a step-by-step process causing a gradual alteration in the qualitative (germination, flowering, etc.) and quantitative (number of leaves, number of flowers, etc.) phenotype of the plant [1]. After germination and before reproduction, plants go through a vegetative growth phase during which mass and photosynthetic capacity are rapidly increased. The vegetative growth phase consists of a juvenile and an adult phase that are distinguishable by unique growth patterns and structures. During the juvenile vegetative phase, plants are generally insensitive to photoperiod and floral inducers, and with the transition to the adult vegetative phase, they gradually acquire reproductive competence. During vegetative growth, the vegetative phase change is accompanied by species-specific changes in leaf size and shape, internode length, and trichome distribution, ultimately causing a change in the stem appearance, a condition known as heteroblasty [2,3,4]. Phenotypic changes associated with vegetative phase change can be subtle modifications to leaf morphology, such as those observed in certain annual species (e.g., maize [5] and Arabidopsis [6]), or much more dramatic changes affecting the whole structure of the shoots in perennials like *Acacia, Eucalyptus*, *Quercus*, and *Hedera species* [7]. During the reproductive phase transition, plants switch from vegetative to reproductive growth and the vegetative shoot apical meristem converts into an inflorescence meristem [8]. Most of the changes associated with vegetative to reproductive phase transition in annual plants are unidirectional; that is, once the plants enter the adult vegetative phase, they continue forward with the reproductive phase. Perennial plants, however, alternate between the adult vegetative and the reproductive phases [9,10]. Most perennial species have a polycarpic growth habit, and they undergo many reproductive cycles during their lifetimes. In a perennial plant, different meristems exhibit different behaviors so that some undergo floral transition while others remain in the vegetative state [11,12,13].

A secondary phase change, when a reversion from the adult to the juvenile phase occurs following pruning, grafting or tissue culture, allows plants to restore juvenile features [14]. The reversibility of phase change has led to a long-standing view that epigenetics plays a major role in its regulation [15]. In this paper, we discuss the physiological, epigenetic, and genetic control of vegetative phase change and rejuvenation in plants.

## 2. The Role of Endogenous Factors in Vegetative Phase Change and Rejuvenation

Endogenous factors have significant roles in vegetative phase change and rejuvenation. Hormones play crucial roles in the rejuvenation of trees, with cytokinins and gibberellins able to induce rejuvenation and maintain the juvenile state [16,17]. Huang et al. [18] showed that the ability to root in successive generations of cuttings of the shrub *Buxus sinica* var. *parvifolia* was correlated with changes in the concentrations of hormones such as indole-3-acetic acid (IAA), abscisic acid (ABA), and gibberellin A4 (GA4). The IAA/ABA content determines the degree of the juvenile phenotype in vitro and the rooting capacity of tender stems. In the species annual *Arabidopsis thaliana*, glycine-rich RNA-binding proteins gather at different stages of rejuvenation and affect the recovery of rooting capacity which is regulated by both auxin and ABA [19,20]. 

The effects of GA in controlling phase change depend upon the species. Exogenous GA application was found to promote rejuvenation in English ivy [21] and in *Acacia melanoxylon* [22], while it accelerated vegetative phase change and flowering in maize [23] and *Arabidopsis* [6,24]. In some perennial species, GA can cause a reversion of the reproductive phase to the vegetative phase [25]. The concentrations of IAA and ABA were found to be higher in rejuvenated shoots than in mature walnut shoots, while GA3 and zeatin-riboside (ZR) showed the opposite pattern [26]. In *Sequoia sempervirens*, genes regulating phytohormones are the targets of small RNA (sRNA). One of them is the gene specific for ABA biosynthesis enzyme (9-cis-epoxycarotenoid dioxygenase) that has a lower expression in adult shoots compared to juvenile and rejuvenated shoots. ABA content increases up to 1000-fold during shoot maturation, while it decreases considerably during in vitro plant rejuvenation [27]. Such increase in ABA content might be the result of sRNA regulation, transcriptional activation, or reduced ABA turnover [27]. Additionally, three genes in Yang’s cycle (in the ethylene biosynthesis cycle) were also found to be highly expressed in adult shoots compared with rejuvenated or juvenile shoots when there was no intervention by sRNAs, indicating the role of ethylene in phase transitions [27].

Rejuvenation includes restoring juvenile features, such as increasing esterase and peroxidase activities [28] and improving photosynthetic and respiratory rates [29]. Phase reversal is also accompanied by genomic features such as a rearrangement of nuclear and mitochondrial DNA, restoration of protein phosphorylation or phosphokinase activity, a global decrease in nuclear DNA methylation and an increase in mitochondrial DNA methylation [30], reappearance of mitochondrial circular DNA molecules [31], and the recovery of sRNA expression [27]. 

Global changes in DNA methylation have been associated with tissue development and differentiation as well as organ function acquirement [32]. In the heterophyllous species *Ilex aquifolium*, change in leaf morphology induced by herbivory pressure, from entire adult leaves to dentate juvenile leaves, correlates with change in leaf DNA methylation profiles [33]. Callus-induced dedifferentiated cells were found to have higher levels of euchromatin (open chromatin) in comparison to differentiated cells that are richer in heterochromatin (closed chromatin) in Arabidopsis [34]. Gene expression ontology studies of in vitro culture-induced citrus callus, overexpressing the microRNA *miR156*, identified DNA methylation processes as enriched during culture [35]. Mobile sRNAs, which have been shown to be part of a systemic signaling pathway in plants [36,37,38,39,40], have also been shown to be capable of modifying the methylation profiles of the sink organs they target in many species. Taken collectively, these results indicate that multiple molecular mechanisms interact to regulate epigenetic profiles at phase transitions.

## 3. Vegetative Phase Change Control in Annual Species and Woody Perennials

The timing of the transition between the juvenile to adult phases differs hugely depending upon the species. In annual species, this transition happens relatively soon after the completion of germination, while in perennials, this transition might take months or years. Perennials also exhibit major morphological changes in shoot architecture before and after vegetative phase change compared to annuals. The morphological changes associated with vegetative phase change in perennials need to be temporally and spatially coordinated, as these species encounter environmental constraints depending on season and many biotic and abiotic stresses during their long lifecycles [41]. In studies on annuals such as *Arabidopsis thaliana* and maize (*Zea mays),* the microRNAs (miRNA) *miR156* and *miR172* have been found to regulate phase transitions [42,43]. In annual species, the expression of *miR156* is very high in the seedling stage and gradually declines with the juvenile-to-adult transition, while *miR172* shows the opposite expression pattern [7]. A similar miRNA expression pattern has also been observed in perennial woody species with highly characterized juvenile and adult phases such as *Acacia confusa*, *Acacia colei*, *Eucalyptus globulus*, *Hedera helix*, and *Quercus acutissima* [7]. *miR156* is common to almost all the major plant taxa and its role in the control of vegetative phase change seems to be conserved throughout the whole plant kingdom [7].

## 4. miRNAs Regulate Vegetative Phase Change Genes

Vegetative phase change is regulated by the post-transcriptional repression of phase change genes by specific, non-coding miRNA families, and these miRNA encoding genes are themselves epigenetically regulated. Increased accumulation of *miR156* and *miR157* delays the juvenile to adult transition, while accumulation of *miR172* and *miRNA159* accelerates this transition [42,44]. Although phenotypic alterations associated with vegetative phase transition are very distinct between annual and perennial plants, vegetative phase change in both is controlled by the same miRNA families. 

In annual plants such as maize and *Arabidopsis*, molecular genetic analyses showed that *miR156* plays a crucial role in vegetative phase change [45,46]. During the juvenile phase, the expression of *miR156* is elevated and it declines during vegetative phase change. The targets of *miR156* are *SQUAMOSA PROMOTER BINDING PROTEIN*-*like* (*SBP/SPL*) genes [47,48,49]. SPL is a plant-specific transcription factor family, first discovered in *Antirrhinum majus* by Klein et al. [50], that exists in all plant taxa studied. Many *SBP/SPL* genes are regulated by *miR156* [51,52,53], and *miR156*-regulated *SPL* genes are believed to control similar sets of traits in different plant species, as the phenotypes of the plants overexpressing *miR156*-encoding genes are almost identical in all species studied [45,46,52,54]. SPL transcription factors vary in size, ranging from 100 to 927 amino acids in *A. thaliana* [55], and include an evolutionarily conserved DNA binding domain that is around 76 amino acids long [51]. They regulate many important parts of a plant’s life cycle including vegetative phase change, inflorescence architecture, fruit development, grain morphology, leaf initiation, and pollen development [56,57]. *SPL* genes in *A. thaliana* carry a *miR156* microRNA response element within their 3’-UTR region that is highly complementary to *miR156* [47]. *miR156* causes transcriptional repression through cleavage of SPL transcripts, resulting in reduced SPL mRNA levels [46,52]. As *miR156* levels decrease with aging, this leads to an increase in *SPL* transcripts which results in the initiation of phase transition. 

*miR172* is also known for its involvement in vegetative phase change and flowering and was first identified in Arabidopsis [58]. *miR172* targets the mRNA of many transcription factors associated with the APETALA2 (AP2)-like protein, including Glossy15 (GL15) in maize [43]. The GL15 transcription factor maintains the juvenile state, increasing the number of juvenile leaves and delaying the flowering process. *miR172* regulates the phase transition by cleavage and negative regulation of *GL15* [43]. *miR172* levels start to increase after germination and continue to increase gradually with plant maturation, the opposite of the *miR156* expression pattern. Overexpression of *miR156* extends the expression of juvenile vegetative traits and delays flowering [45,46], whereas the overexpression of *miR172* accelerates flowering [59,60,61]. *miR156* negatively regulates *miR172* activity [62] by targeting SPL transcription factors, which are positive regulators of *miR172* expression [42,63]. As the plant matures and *miR156* transcription declines, higher levels of *miR172* eventually lead to the downregulation of *GL15* [43]. Overexpression of *miR156* in the perennial *Populus × canadensis* also downregulated the expression of *SPL* genes and *miR172* and extended the juvenile phase [7].

Additionally, *miR159* plays an essential role determining the correct timing of juvenile-to-adult phase transition during vegetative development by blocking the expression of *miR156* [64]. *miR159* is highly conserved and abundant throughout land plants and targets a class of genes encoding for R2R3 MYB domain transcription factors [65]. Guo et al. [64] showed that the loss of the re-pressive effect of *miR159* on *miR156* results in a delay of the juvenile-to-adult transition while the overexpression of *miR159* quickens such transition. *miR159* acts as a molecular switch to silence MYB33 [66], which is responsible for promoting the transcription of MIR156A, MIR156C, and SPL9 simultaneously through binding to their promoters [64]. Recent work on *VIVIPAROUS/ABI3-LIKE (VAL)* gene family suggested their influence on vegetative phase transition. *VAL* genes are well known for regulating other developmental transitions, such as seed maturation [67] and flowering [68], which made them an excellent candidate to further investigate their influence on *miR156* and vegetative phase change. In fact, a study conducted by Fouracre et al. [69] on the epigenetic repression of *miR156* revealed that *VAL1* and *VAL2* genes critically and redundantly regulate the levels but not the temporal patterns of *miR156*. The authors also reported that in addition to the expected miR156-dependent pathway, *VAL* genes regulate plant vegetative phase change via an miR156-independent mechanism [69]. Interestingly, Yu et al. [70] have shown that, besides the regulatory effects of miRNAs 172 and 159 on *MIR156*, there is a third endogenous factor affecting vegetative phase transition through the regulation of *miR156*. The authors showed that the gradual decline of *miR156* with plant age correlates with an accumulation of sugars. Importantly, by using photosynthetic mutants and defoliation assays, they showed that sugar accumulation leads to the transcriptional and post-transcriptional repression of *miR156* and to the initiation of the vegetative phase transition [70].

## 5. The Influence of Histone Modifications in Phase Transition

The *miR156/157-SPL* pathway is the master regulator of vegetative phase change in plants, and the genes that are associated with this pathway undergo epigenetic regulation via histone modification and chromatin remodeling. Alteration of chromatin structure is a prerequisite for the downregulation of *MIR156A* and *MIR156C* and the expression of genes encoding these miRNAs is regulated by the chromatin modification polycomb repression complex 2 (PRC2)-mediated histone H3 lysine 27 trimethylation (H3K27me3). H3K27me3 is a repressive chromatin mark and a major silencing mechanism in plants with a crucial role in regulating the timing of developmental phase transitions [71,72,73]. It downregulates embryonic genes from the roots and shoots and represses *SHOOTMERISTEMLESS* (*STM*) in leaves. H3K27me3 also has a contribution in controlling flowering time by preventing the early expression of floral genes [74] and repressing *FLOWERING LOCUS C* (*FLC*). Whole-genome analysis showed that there are thousands of loci in the *A. thaliana* genome carrying the H3K27me3 mark catalyzed by the PRC2 complex [75,76]. Many *MIR156/157* loci (specifically the dominant loci *MIR156A*, *MIR156C*, and *MIR157A*) have H3K27me3 marks. *SPL* genes responsible for juvenile-to-adult transition lack the H3K27me3 mark, suggesting that PRC2 promotes the transcription of the *SPL* genes by suppressing the transcription of *MIR156/157* loci [76]. During vegetative phase change, the reduction in overall transcription of *MIR156A* and *MIR156C* loci is due to an increase in binding of the PRC2 complex, which ultimately leads to an increase in the H3K27me3 mark in their promoters and transcribed regions and a decrease in the H3K27 acetylation mark close to transcription start sites [77]. Mutations in the chromatin-remodeling complex SWR1 and the genes encoding H2A.Z also cause a significant reduction in the expression of *MIR156A* and *MIR156C* which leads to the acceleration of vegetative phase change. H2A.Z promotes *MIR156A* and *MIR156C* expression in the early, juvenile stage by aiding the deposition of an alternative lysine methylation mark, H3K4me3 [77]. In each generation, the *miR156/157* silencing mechanism is reset back to the active state [78].

## 6. The Reversibility of Phase Change—Rejuvenation and Regrowth

Under certain circumstances, phase change is reversible. This secondary phase change, when plants go back from the adult phase to the juvenile phase and shoot meristems attain juvenility, is also known as rejuvenation (i.e., plants regain juvenile physiological features) [79]. Plant rejuvenation can be induced through severe pruning, in vitro tissue culture, and in vitro repetitive grafting of mature shoot tips onto juvenile rootstocks.

*miR156*, the master regulator of vegetative phase change, also appears to have a crucial role in plant rejuvenation events. Studies on in vitro maize culture have shown that *miR156* levels are significantly increased in adult shoot apices [80] and that the expression of *SPL* genes is significantly lower in rejuvenated maize shoots [81], indicating that the *miR156-SPL* pathway might play a role in plant rejuvenation. The role of sRNAs has also been studied in perennials. Comparison of the expression profiles and target gene prediction of sRNAs in juvenile, adult, and rejuvenated *S. sempervirens* identified some unique sRNAs with possible functions in controlling photosynthesis and rooting competence during plant rejuvenation [27]. An increase in *SsmiR156* and a decrease in *SsmiR172* was found in the rejuvenated plants, suggesting they might have a role in reversing vegetative phase change in addition to their canonical role during plant phase transition. However, while overexpression of *miR156* in *A. thaliana* extends the expression of juvenile traits such as juvenile leaf characters, higher leaf initiation rates, increased branching density, and flowering delay [42] in juvenile plants, recent work on *A. thaliana* by Ye et al. [82] revealed that *miR156* alone is not able to fully induce plant rejuvenation in adult plants. In their work, the authors suggest two plausible explanations for this observation. First, in adult plants, the epigenetic state of genes regulated by SPL is mitotically stable and irreversible, even when *miR156 is* overexpressed. Alternatively, they proposed the existence of an unknown plant aging pathway which is dominant over the effects of *miRNA156*.

## 7. Environment−Epigenetic Interactions Regulating Phase Transition and Regrowth

Plants must constantly adapt to changing environmental conditions to survive, and they have sophisticated mechanisms to regulate important genes in response to environmental fluctuations. Plants can sense environmental signals and transmit those signals using signal transduction. This triggers a cascade of chemical reactions and the accumulation of required transcription factors that activate the genes necessary for survival and adaptation [83].

**Figure 1:**
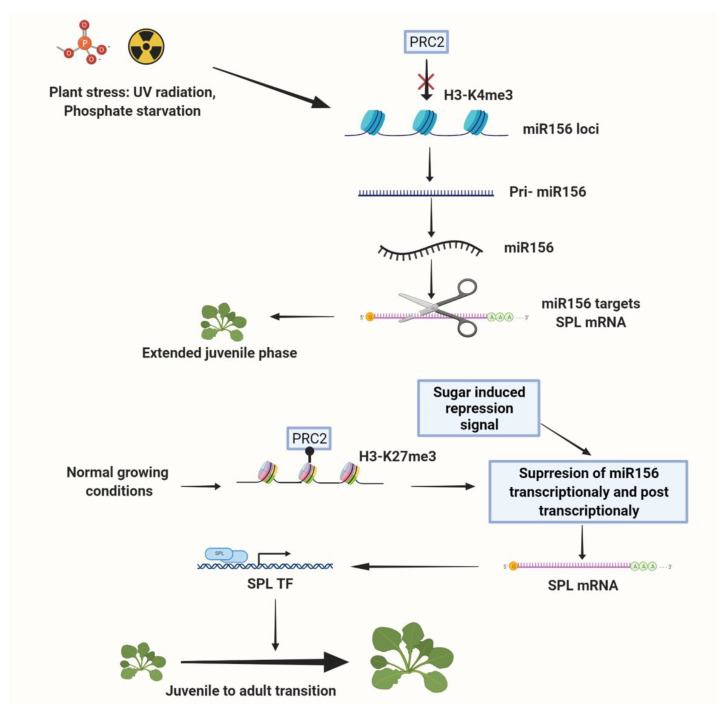
Proposed model for environment−epigenetic interactions regulating vegetative phase transition. Under normal conditions, PRC2-mediated H3K27me3 modification and sugar-induced transcriptional and post-transcriptional *miR156* suppression influence the transition from the juvenile to the vegetative adult phase. Under stresses such as salinity, UV-B radiation, and phosphate starvation, plants experience an extended juvenile phase because of a reduction in PRC2-mediated H3K27me3 modification at *MIR156A/MIR156C* loci.

Under abiotic stress, one important strategy that plants use to survive and adapt is controlling phase transition to either prolong or shorten the length of their juvenile phase and adjust their flowering time. Under certain unfavorable environmental conditions such as drought stress (under short days), salt stress [84], or phosphate starvation [85], the expression of *miR156*-encoding genes is induced to maintain the juvenile phase of the plant for a comparatively longer period. Under UV-B radiation, *miR156* is upregulated by the reduction in PRC2-mediated H3K27me3 modification at *MIR156A/MIR156C* loci, similarly resulting in a delay in vegetative phase change [86] (Figure 1). Once the environment returns to favorable conditions, *miR156* is suppressed, and the vegetative phase transition initiated [83]. It is important to highlight that it has also been observed that other stresses (e.g., drought or elevated temperatures) can instead accelerate flowering. In *A. thaliana*, drought induces flowering just after exposure to long day conditions, suggesting that drought-induced flowering requires a previous environmental trigger [87]. In high temperature stress, although *miR156/miR157* expression increases and *miR172* expression is downregulated, *SPL* responses are mixed, with only transient downregulation, and flowering time is advanced [88,89]. Nonetheless, acquired thermotolerance in *A. thaliana*—when plants have heat stress memory and improved tolerance of a recurring heat stress—was shown to depend on this regulation of *miR156-SPL* expression during the first exposure [89]. Thus, the *miR156-SPL* module has been shown to regulate stress tolerance and control the vegetative phase transition in response to environmental stress, mediated by reversible epigenetic modifications (Figure 1).

Sometimes, plants are exposed to extreme stresses such as browsing or fire which result in severe damage to the crown tissue or above-ground parts. Despite after-fire events giving the surviving plants a “window of opportunity” for regeneration when they do not have to compete for the resources, such as light, nutrients, and water [90], a huge number of plants are killed by the total defoliation caused by fire, and only a few of them show the capacity to re-sprout [91]. Re-sprouting can start from above- or below-ground tissues depending on the number and location of dormant buds [92]. Depending on the ecosystem and species, a new plant can emerge after the crown damage of the mother plant from structures such as roots, rhizomes, tubers, lignotubers, or corms. Because of the excellent heat-insulating capacity of the soil, these bud-bearing, below-ground structures provide fitness benefits in a fire-prone ecosystem.

Regrowth can also begin from above-ground structures such as epicormic buds, one type of developmentally arrested, accessory meristem. In a normal situation, their growth is suppressed hormonally, but they are capable of becoming active shoots when the primary shoots are damaged or decapitated [93,94]. Whole plants are able to re-sprout and regrow from these arrested buds that have previously been held in a fixed, juvenile state.

*Eucalyptus* trees are best known for their ability to regenerate branches vegetatively from epicormic buds along their trunks. Epicormic buds of *Eucalyptus* are located much deeper in the bark than in most species, at the level of vascular cambium, and are therefore more protected from fire damage [91]. Epicormic buds remain in an arrested state when the shoot undergoes phase change, indicating that accessory meristems such as epicormic buds do not have the innate timing responses of apical meristems and that they are influenced by the status of the whole plant [92].

In annual *A. thaliana*, *miR156*-targeted *SPL* controls both shoot regeneration [95] and root meristem activity that determines root-derived de novo shoot regeneration [96] in an age-dependent manner. A comparative physiological and molecular analysis between perennial *Arabis alpina* and annual *A. thaliana* also showed that differential expression of *miR156* determined the polycarpic perenniality in *A. Alpina* [97]. These findings, together with the conserved molecular pathway for rejuvenation in woody perennials and annuals, suggest that the *miR156-SPL* pathway, which is in action during plant rejuvenation, might also influence plant regrowth and re-sprouting capacity following severe damage. However, further studies comparing plant rejuvenation and plant resprouting are required to understand their similarities and differences.

## 8. Future Directions

Although much is known about the physiology, genetics, and epigenetics of vegetative phase change, there are many long-standing questions about both vegetative phase change and rejuvenation which remain to be answered. Phase transition is associated with changes in miRNA expression, but how plants detect the correct developmental phase is unknown. In many cases, the source and the identity of the signals that initiate/revert this transition are still elusive [82]. Recent studies have shown that the miRNA families that control vegetative phase change in annuals are also responsible for phase transitions in perennials. In perennials, vegetative phase change is associated with dramatic changes in shoot architecture, while only minor phenotypic changes occur in annuals, and it is not known why perennials have more distinct juvenile and adult-phase phenotypes compared with annuals despite being regulated by the same mechanisms. The expression patterns of *miR156* and *miR157* are similar in rejuvenated and in juvenile shoots [80], but this proposed similarity is still quite obscure, and more comparative studies between these two stages are required to assess how similar they are. Many of these questions may best be answered by using epigenetic analyses as tools to study vegetative phase change and rejuvenation in both annual and perennial systems. All the epigenetic features (DNA methylation in nuclear and mitochondrial DNA, sRNA molecules, and rearrangement of nuclear and mitochondrial DNA) associated with vegetative phase reversal [27,30,31] indicate that they are synchronized to maintain epigenetic memory during vegetative propagation. Further studies and possibly the identification of a plant model suitable for the molecular dissection of plant rejuvenation [82] are needed to unravel the molecular mechanisms of epigenetic memory which will not only aid our understanding of plant rejuvenation and phase transitions but can also be utilized as a tool to allow breeders, nurseries, and growers to maintain favorable traits during clonal propagation.
